# The (pro)renin receptor mediates constitutive PLZF-independent pro-proliferative effects which are inhibited by bafilomycin but not genistein

**DOI:** 10.3892/ijmm.2014.1624

**Published:** 2014-01-14

**Authors:** SEBASTIAN KIRSCH, EVA SCHREZENMEIER, SABRINA KLARE, DANIELA ZAADE, KERSTIN SEIDEL, JENNIFER SCHMITZ, SARAH BERNHARD, DILYARA LAUER, MARK SLACK, PETRA GOLDIN-LANG, THOMAS UNGER, FRANK S. ZOLLMANN, HEIKO FUNKE-KAISER

**Affiliations:** 1 Institute of Laboratory Medicine, Clinical Chemistry and Pathobiochemistry, Charité - Universitätsmedizin Berlin, Germany; 2 Center for Cardiovascular Research (CCR)/Institute of Pharmacology, Charité - Universitätsmedizin Berlin, Berlin, Germany; 3 Evotec AG, Hamburg, Germany; 4 School for Cardiovascular Diseases (CARIM), Maastricht University, Maastricht, The Netherlands

**Keywords:** (pro)renin receptor, small-molecule drug, cancer, PLZF, glucose

## Abstract

The (pro)renin receptor [(P)RR] is crucial for cardio-renal pathophysiology. The distinct molecular mechanisms of this receptor are still incompletely understood. The (P)RR is able to interact with different signalling proteins such as promyelocytic leukemia zinc finger protein (PLZF) and Wnt receptors. Moreover, domains of the (P)RR are essential for V-ATPase activity. V-ATPase- and Wnt-mediated effects imply constitutive, i.e., (pro)renin-independent functions of the (P)RR. Regarding ligand-dependent (P)RR signalling, the role of prorenin glycosylation is currently unknown. Therefore, the aim of this study was to analyse the contribution of constitutive (P)RR activity to its cellular effects and the relevance of prorenin glycosylation on its ligand activity. We were able to demonstrate that high glucose induces (P)RR signal transduction whereas deglycosylation of prorenin abolishes its intrinsic activity in neuronal and epithelial cells. By using siRNA against (P)RR or PLZF as well as the PLZF translocation blocker genistein and the specific V-ATPase inhibitor bafilomycin, we were able to dissect three distinct sub-pathways downstream of the (P)RR. The V-ATPase function is ligand-independently associated with strong pro-proliferative effects whereas prorenin causes moderate proliferation *in vitro*. In contrast, PLZF *per se* [i.e., in the absence of (pro)renin] does not interfere with cell number.

## Introduction

The (pro)renin receptor [(P)RR also called RER] constitutes a novel component of the renin-angiotensin system (RAS) and exerts pivotal functions in cardio-renal pathophysiology since it is linked to angiotensin II-dependent and also angiotensin II-independent effects (1). Binding of renin to the (P)RR increases its catalytic efficiency 4- to 5-fold, whereas binding of prorenin non-proteolytically demasks the catalytic activity of prorenin (2).

Various research studies have demonstrated that the competitive inhibition of the binding of (pro)renin to the (P)RR by parenteral application of a decoy peptide, which is derived from the prosegment of prorenin, prevents the development of diabetic nephropathy and reduces cardiac fibrosis (3–6). Importantly, (pro)renin receptor blockade by these decoys is also effective in angiotensin AT1 receptor (AT1R)-knockout mice (7) and can even reverse renal damage caused by diabetes (8).

Nevertheless, a number of authors were not able to observe positive effects of these peptides (9,10). These controversies may be explained by the prorenin-renin ratio since the decoys were effective in high prorenin/low renin models (1,11), and/or by the length of treatment based on the observation that renoprotective effects were able to be observed after 12 weeks even in a high renin Goldblatt model (12,13).

In addition, the effectiveness of the decoy peptides regarding reduction in weight gain, serum triglycerides and hyperinsulinemia has been shown in animals fed with a high fat/high carbohydrate diet (14,15). Beneficial effects were also observed concerning diabetic retinopathy (16–18). Recently, it was demonstrated that the (P)RR was upregulated at the mRNA and protein levels in murine hearts after myocardial infarction as well as in patients with dilated cardiomyopathy (19) further supporting the pathophysiological importance of this receptor. Consistently, at the molecular level, activation of the (P)RR by (pro)renin is associated with a detrimental transcriptional signature, e.g. linked to cardiac hypertrophy, cardiac and renal cell death (20). Moreover, the (P)RR is upregulated on the mRNA and protein levels in the hearts and kidneys of diabetic rats (21,22).

Our research group revealed a novel signal transduction pathway involving the physical interaction between the (P)RR and the transcription factor promyelocytic leukemia zinc finger protein (PLZF). Upon stimulation of the (P)RR with renin or prorenin, PLZF was found to translocate into the nucleus and repress the RER promoter itself (23,24). Regarding the ligand level, different renin glyocoforms, which are likely linked to the differential plasma half-lives, have been previously described (25).

In addition, renin-independent functions of the (P)RR have been recently described (26). In this context, it is important to note that the (P)RR protein consists of an evolutionarily conserved part (comprising the C-terminal 69–100 amino acids) and a large N-terminal (pro)renin binding domain (1,27). The ancient part is identical to the vacuolar proton-translocating ATPase (V-ATPase) membrane sector-associated protein M8–9 (28,29). Therefore, it has been suggested that the gene encoding the (P)RR results from a gene fusion (30). Nevertheless, these parts can be separated at the protein level again based on the identification of a soluble (P)RR isoform [s(P)RR] which nearly represents the extracellular part (31). The s(P)RR is generated by the action of furin and/or ADAM proteases (31,32).

Considering the different protein isoforms of the (P)RR, it has been shown that the specific V-ATPase inhibitor bafilomycin A1 inhibits (P)RR signalling (29). In addition, the nuclear translocation of PLZF, in the context of the angiotensin AT2 receptor (AT2R), is inhibited by the small molecule genistein (33). Currently, genistein and bafilomycin are the only drug-like, commercially available small molecules directly interfering with (P)RR signal transduction.

Therefore, the aim of the present study was to analyse (pro)renin-independent, i.e., constitutive, activity of the (P)RR, the transcriptional and isoform-specific regulation of this receptor as well as the effects of genistein and bafilomycin on its signal transduction. Since constitutive receptor activity does not exclude ligand effects (34) and may be unveiled by inactivating post-translational modifications of the ligand, we also analysed the effects of prorenin deglycosylation.

## Materials and methods

### Cell culture

B-16V (mouse melanoma) and KELLY (human neuroblastoma) cells (both from DSMZ, Braunschweig, Germany) were grown in RPMI-1640 medium (Life Technologies, Darmstadt, Germany). HeLa-S3 (human cervical carcinoma), HEK293 (human embryonic kidney) (both from DSMZ) and HEK293T cells [American Type Culture Collection (ATCC), Manassas, VA, USA] were cultivated in DMEM high glucose (Life Technologies). Flp-In-293 HEK cells (Invitrogen, Karlsruhe, Germany) were grown in DMEM high glucose supplemented with 2 mM glutamine (PAA, Pasching, Austria). All media contained 10% fetal bovine serum (Life Technologies), 100 U/ml penicillin and 100 μg/ml streptomycin (A2212; Biochrom, Berlin, Germany). If the experimental conditions were modified the changes are described in the Results section. All cell lines were cultivated without addition of an angiotensin AT1 receptor blocker (ARB) and, if not otherwise specified, without starving in a humidified incubator with 5% CO_2_ at 37°C.

All experiments using the stably transfected HeLa cell line were performed in 48-well plates (BD Falcon, Franklin Lakes, NJ, USA). For all transient transfections of (P)RR promoter constructs and (P)RR expression vectors, 24-well plates (BD Falcon; from Corning Inc., Corning, NY, USA) (in the case of KELLY cells) and 6-well plates (BD Falcon), respectively, were used. Prorenin was obtained from Innovative Research (Novi, MI, USA). Bafilomycin A1 (Enzo Life Science, Lörrach, Germany) and genistein (Carl Roth GmbH, Karlsruhe, Germany) were dissolved in 1% DMSO final if not otherwise stated.

### Subcloning and transient transfection experiments

The full-length human (P)RR coding sequence was cloned into pEGFP-N1 and pEGFP-C3 vectors (Clontech, Mountain View, CA, USA) as described previously (23). A construct containing 1,100 bp (directly upstream of the translational start site) of the human (P)RR was subcloned into the pGL4.14 luciferase vector (Promega, Mannheim, Germany) using previously published primers (23). Transient transfection experiments of these expression vectors were perfomed using Genejuice (Merck, Darmstadt, Germany) or Turbofect (Fermentas, St. Leon-Rot, Germany) transfection reagents according to manufacturers’ protocols with 25 ng DNA/cm^2^.

SiRNA experiments were performed with siRNA against (P)RR [5′-gcuccguaaucgccuguuu-3′ (sense strand); 20 nM final] or scrambled control siRNA [5′-uuuaccgucgccuugagcu-3′ (sense strand)] (Eurogentec, Köln, Germany) and against PLZF [5′-ccagcaagauguuugagau-3′ (sense strand); 50 nM] or scrambled control siRNA [5′-ucucgcagugacuauacau-3′ (sense strand)] (Eurogentec), respectively, using HiPerfect (Qiagen, Hilden, Germany). General efficacy of siRNA-mediated knockdown was controlled in KELLY cells by real-time polymerase chain reaction (PCR) ((P)RR mRNA was decreased to 10–30% relative to the scrambled control) and Western blotting (RER protein was decreased to ~40% relative to the scrambled control) and in double-stably transfected HeLa cells by Western blotting.

### Generation of stable cell lines

To measure the activity of the (P)RR, a double-stable, double-monoclonal HeLa cell line was generated using the human (P)RR promoter/pGL4.14 (firefly) and pGL4.79 (*Renilla*) plasmids (Promega). The (P) RR promoter sequence and the assay principle were based on Schefe *et al* (23) and on a patent application of our group (EP 1 890 152 A1 or PCT WO 2008/019735 A9). The *Renilla* luciferase activity served for standardisation. After the first transfection (pGL4.14) cells were selected using hygromycin B (250 μg/ml medium; PAA) and monoclonalised using cloning cylinders (C7983; Sigma-Aldrich, Steinheim, Germany). After the second transfection (pGL4.79) selection was performed by addition of G-418 sulphate (500 μg/ml medium; PAA) followed by monoclonalisation.

A prorenin-overexpressing and prorenin-secreting HEK293 cell line was generated using the Flp-In system (Invitrogen) and an expression vector (pcDNA5/FRT) encoding preprorenin fused to a C-terminal His_10_-tag.

### Fractionated protein extraction

Nuclear and cytosolic proteins were isolated as described previously (35). Nuclear fractions were controlled by Wsestern blotting using an antibody against TFIID as described below.

### Protein purification and prorenin deglycosylation

His_10_-tagged prorenin was purified via metal affinity chromatography as follows. Flp-In-293-HEK cells stably transfected with an expression vector encoding C-terminally His_10_-tagged preprorenin were cultured without starving in 1 or 4.5 g/l glucose concentrations for two weeks followed by a two-day serum-free period to exclude interference of serum proteins before collection of the supernatant for affinity chromatography of prorenin. A gravity column (Empty Disposable PD-10 Column, 17-0435-01; GE Healthcare, Munich, Germany) was loaded with 5 ml of a cobalt matrix (Talon Metal Affinity Resin; Clontech, Saint-Germain-en-Laye, France) and equilibrated with 20 ml washing buffer (50 mM sodium dihydrogen phosphate, 300 mM sodium chloride, pH 8.0). Two hundred milliliters of the cell culture supernatant was loaded on the column. The flow-through was discarded. The matrix was washed with washing buffer containing 10 mM imidazole (Sigma-Aldrich). Afterwards, prorenin was eluted with the same buffer but with 250 mM imidazole. Ten fractions each 2 ml were collected and analysed by sodium dodecyl sulfate-polyacrylamide gel electrophoresis (SDS-PAGE) and Coumassie staining. The pure, prorenin-containing fractions were pooled and dialysed in 3 liters of phosphate-buffered saline (PBS) overnight at 4°C. Finally, the protein solution was concentrated using filter devices excluding proteins <30 kDa (Amicon Ultra 0.5 ml; Millipore GmbH, Schwalbach, Germany). The concentration was determined with UV absortion spectrometry (ND-1000; PeqLab, Erlangen, Germany).

Purified prorenin and a commercially obtained prorenin (from Innovative Reseach) were deglycosylated using N-glycosidase F (11365185001; Roche, Mannheim, Germany), which does not exhibit proteolytic activity, for 2 h and resolved via SDS-PAGE followed by Coomassie staining.

### Real-time PCR

Reverse transcription was performed using M-MLV reverse transcriptase (RNase H minus; Promega) and 1 μg RNA. PCR was performed applying Go-Taq qPCR Master Mix (Promega) and the following primer pairs: 5′-ATTGGC CTATACCAGGAGAG-3′ (forward) and 5′-TTCCCCATAAC GCTTCCCAA-3′ (reverse) for (P)RR and 5′-CCGCAGCTAGG AATAATGGAATA-3′ (forward) and 5′-TCTAGCGGCGCA ATACGAAT-3′ (reverse) for 18S rRNA. A reaction without addition of reverse transcriptase served as the negative control. The PCR reactions were run on a Stratagene Mx3000P (Stratagene, La Jolla, CA, USA).

### Western blotting

Immunoblotting was performed as previously described (36) but using a cell lysis buffer containing 1× PBS (pH 7.2, without calcium and magnesium; Invitrogen), 1% Nonidet P-40, 0.5% sodium deoxycholate, 0.1% sodium dodecyl sulfate (SDS) (all from Sigma-Aldrich) and Complete EDTA-free cocktail tablets (Roche). The following primary antibodies were used: anti-PLZF (ab39354; Abcam, Cambridge, UK), anti-GFP (sc-8334; Santa Cruz Biotechnology, Inc., Heidelberg, Germany), anti-TFIID [TATA box-binding protein (TBP)] (sc-273; Santa Cruz Biotechnology, Inc.), anti-(P)RR (ATP6AP2; HPA003156; Sigma-Aldrich), anti-actin (sc-1615; Santa Cruz Biotechnology, Inc.) and anti-GAPDH (MAB374, Chemicon/Merck Millipore, Billerica, MA, USA). Detection of the horseradish peroxidase (HRP)-labelled secondary antibody was performed with an enhanced chemiluminescence (ECL) reagent containing a 1:1 mixture of solution A [100 mM Tris base (pH 8.5), 2.5 mM luminol (Sigma-Aldrich), 0.4 mM *p*-coumaric acid (Sigma-Aldrich)] and solution B [100 mM Tris base (pH 8.5), 0.02% H_2_O_2_]. Proteins were quantified using a Bradford assay (Roti-Nanoquant; Carl Roth). Cell culture supernatants were concentrated using Centriprep^®^ 10K columns followed by Amicon Ultra 3K columns (both obtained from Merck Millipore, Darmstadt, Germany) before Western blotting. Densitometric analysis was carried out using ImageJ 1.42q software (National Institutes of Health, USA).

### Reporter gene assays

Promoter reporter assays regarding stable and transient transfections were performed using the Dual-Luciferase Reporter^®^ assay system (Promega).

For transient transfections, relative luciferase activity (RLA), defined as the mean value of the firefly luciferase/*Renilla* luciferase ratios of each construct related to the insertless reporter plasmid pGL4.14, served as read-out. Regarding the stable transfection, the firefly/*Renilla* ratio served as read-out.

### Cellular phenotypic assays

Cellular proliferation was measured using the BrdU colorimetric cell proliferation ELISA (Roche). Mitochondrial dehydrogenase activity was determined via the Cell Proliferation Assay XTT (AppliChem, Darmstadt, Germany). Cellular ATP concentrations were analysed using the CellTiter-Glo Luminescent Cell Viability Assay (Promega). The Bradford assay was obtained commercially (Roti-Nanoquant; Carl Roth).

To measure lactate dehydrogenase (LDH) activity in the cell culture supernatant, 50 μl medium, 200 μl NADH buffer [0.2 mM β-nicotinamide adenine dinucleotide (reduced disodium salt hydrate; Sigma-Aldrich), 0.1 M potassium hydrogen phosphate buffer (pH 7.4)] and, to start the reaction, 25 μl pyruvate buffer [22.7 mM sodium pyruvate (Sigma-Aldrich), 0.1 M potassium hydrogen phosphate buffer (pH 7.4)] were mixed. After 10 sec, NADH was quantified photometrically at 340 nM (10 times every 10 sec) in a 96-well plate reader.

For the staining of acidic cell organelles (e.g. lysosomes or peroxisomes) HeLa cells were seeded 24 h before stimulation in black 96-well plates (Cellstar MicroClear; Greiner, Frickenhausen, Germany). After 1 h prestimulation with genistein or bafilomycin A1, the dye (LysoTracker Red DND-99; Life Technologies) was added to the culture medium (75 nM final) for 1 h. After replacement of the supernatant by serum- and phenol red-free medium, measurement was carried out according to the manufacturer’s instructions using a fluorescence plate reader (Mithras 940; Berthold, Bad Wildbad, Germany).

### Statistical analysis

For comparisons of two interventions including siRNA effects under DMSO control (Fig. 2A) a two-tailed, unpaired t-test was applied. Regarding multiple comparisons, a one-way analysis of variance (ANOVA) with Bonferroni post-hoc adjustment was performed. Statistical significance was assumed at p<0.05 for t-test and ANOVA. Concerning plotting of the dose-response curves, a sigmoidal regression analysis was used.

## Results

### Effects of genistein, bafilomycin and siRNA interventions on (P)RR promoter activity and (P)RR expression

**Figure 1:**
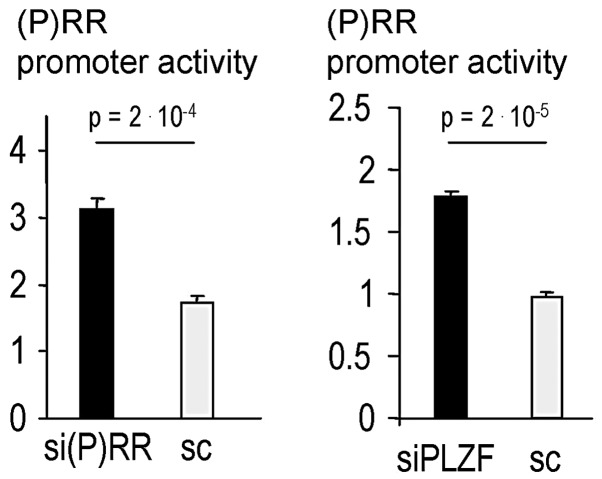
Effects of siRNA against promyelocytic leukemia zinc finger protein (PLZF) or the (pro)renin receptor [(P)RR] on (P)RR promoter activity. HeLa cells double-stably transfected with the (P)RR promoter luciferase constructs were transfected with siRNA against (P)RR [si(P)RR] or against PLZF (siPLZF) (n=3 for each intervention), respectively. Scrambled siRNA (sc) served as the control. Cells were cultured in 4.5 g/l glucose without (pro)renin coincubation.

**Figure 2:**
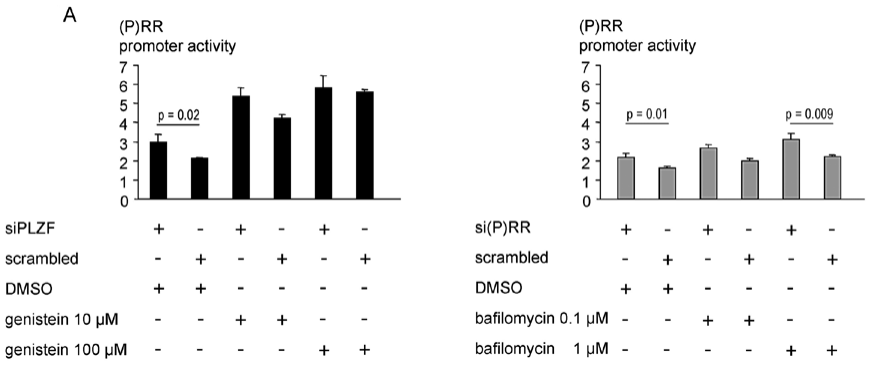

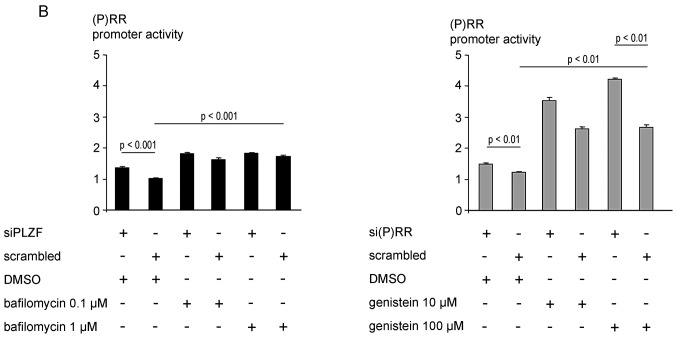

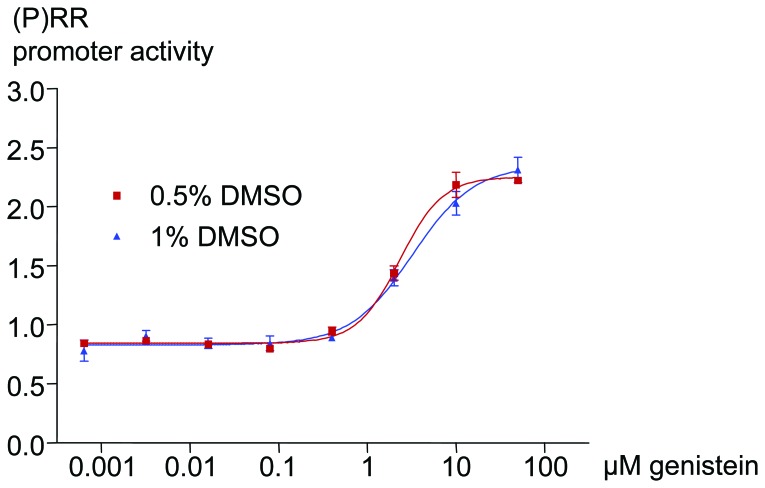

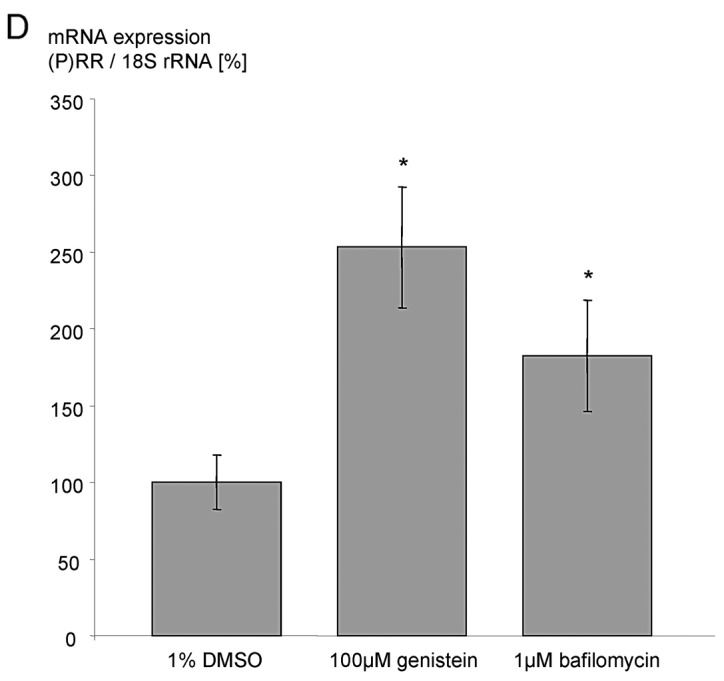

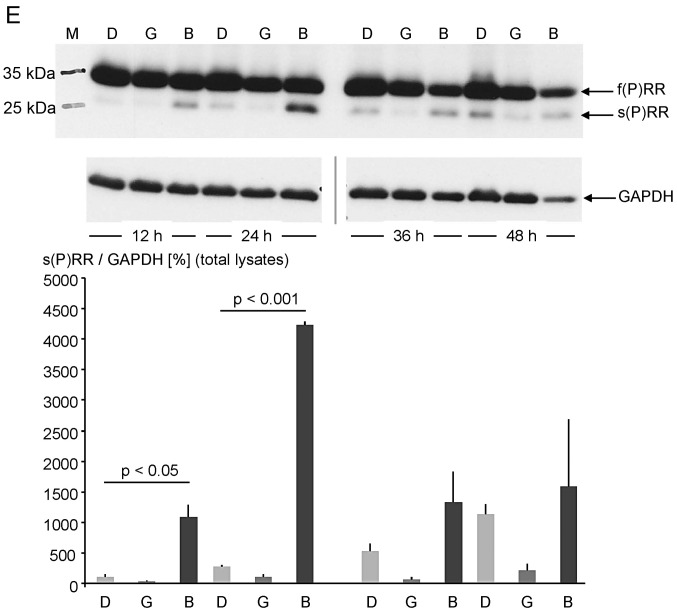

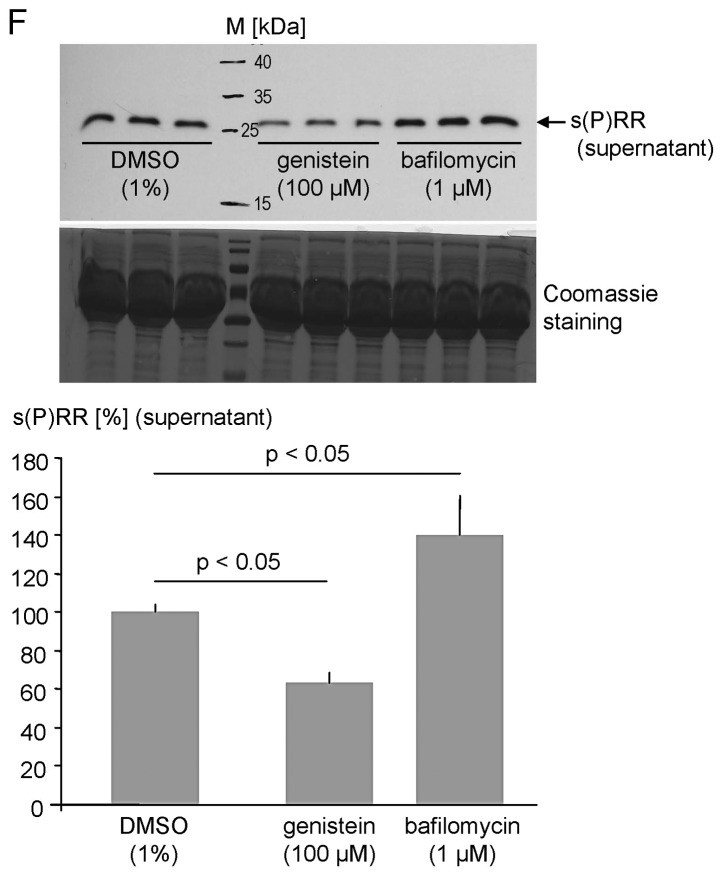
Effects of genistein, bafilomycin and siRNA against promyelocytic leukemia zinc finger protein (PLZF) or the prorenin receptor [(P)RR] on (P)RR promoter activity and mRNA levels. (A and B) HeLa cells double-stably transfected with (P)RR promoter luciferase constructs were cultured in 4.5 g/l glucose (n=3 for each intervention). Incubation times were 48 h for the siRNAs, and 24 h for genistein and bafilomycin. (C) Dose-response curve of genistein in double-stably transfected HeLa cells. (D) Double-stably transfected HeLa cells were treated with genistein or bafilomycin for 12 h followed by real-time polymerase chain reaction (PCR). Data represent the mean expression level of (P)RR mRNA standardised to 18S rRNA expression (± standard deviation) of three independent measurements per cDNA (technical triplicates) calculated according to the ΔΔCt method. The expression ratio of DMSO was set to 100%. ^*^p<0.05 according to ANOVA. (E) Double-stably transfected HeLa cells were treated with genistein (100 nM; G) or bafilomycin (1 μM; B) for the indicated time periods followed by Western blotting of the cellular lysates. f(P)RR, full-lengh (P)RR; s(P)RR, soluble (P)RR. (Lower panel) Densitometric analysis of s(P)RR [mean ± standard deviation of the technical duplicates (one of these shown in the upper panel)] with the ordinate standardised to the GAPDH and normalised to the DMSO control (D) at each point of time is shown. (F) Double-stably transfected HeLa cells were treated with genistein or bafilomycin for 48 h before harvest and concentration of the cellular supernatant followed by anti-(P)RR Western blotting. Coomassie staining served as loading control for supernatant proteins. (Lower panel) Densitometric analysis (mean ± standard deviation of technical triplicates) of s(P)RR in supernatant is shown. M, molecular weight marker.

We previously demonstrated that PLZF is a crucial adapter protein of the (P)RR and its own promoter (23). Other studies have shown that the nuclear translocation of PLZF is inhibited by genistein (33) and that bafilomycin reduces (P)RR signal transduction (29). Therefore, we aimed to ascertain whether genistein, bafilomycin or siRNA against pathway components affects (P)RR promoter activity. As expected, siRNA knockdown of (P)RR and PLZF both significantly derepressed (P)RR promoter activity (Fig. 1). In addition, genistein increased (P)RR promoter activity, whereas bafilomycin had a minor effect (Fig. 2A and B). However, ANOVA using the pooled data shown in Fig. 2A and B indicated that both bafilomycin concentrations significantly increased promoter activity (p<0.01 for 0.1 μM and p<0.001 for 1 μM). Moreover, siRNA against (P)RR and also PLZF caused a derepression of (P)RR promoter activity in the context of coincubations (Fig. 2A) consistent with a repressive role of the (P)RR-PLZF pathway on the (P)RR promoter (23). Importantly, the effect of siRNA against PLZF was abolished by genistein (Fig. 2A) and bafilomycin (Fig. 2B), whereas both substances did not inhibit the effect of siRNA against (P)RR (Fig. 2A and B).

In addition, the EC_50_ value of genistein was determined showing a potency of 2–4 μM regarding promoter activation (Fig. 2C). In addition, a non-sigmoidal concentration-response relationship was also observed concerning the effects of bafilomycin on (P)RR promoter activity (data not shown).

Finally, we analysed whether the effects of genistein and bafilomycin can also be observed at the endogenous transcript level. Both interventions significantly increased (P)RR mRNA after 12 h (Fig. 2D) when effects on cell number are unlikely.

Additionally, the effects of genistein and bafilomycin on the protein level were analysed with respect to full-length and soluble (P)RR. After 12 and 24 h of incubation and upon examination of the total cellular lysates, treatment with bafilomycin but not genistein significantly increased soluble (P)RR (Fig. 2E). Consistently, a significantly increased s(P)RR level was observed in the cellular supernatant following incubation with bafilomycin (Fig. 2F).

In this context, it is important to note that the antibody used in Fig. 2E and F only detects the N-terminal part of the (P)RR, i.e., full-length and s(P)RR, but not the V-ATPase-associated isoform.

### Effects of glucose on (P)RR promoter activity and (P)RR isoform expression

**Figure 3:**
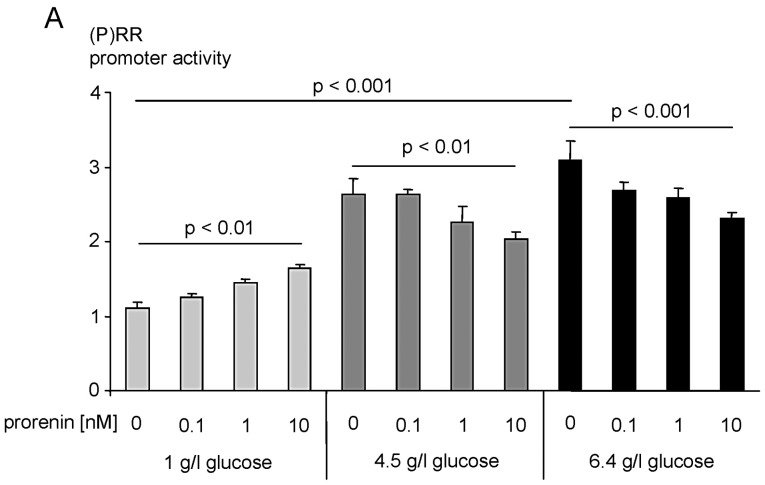

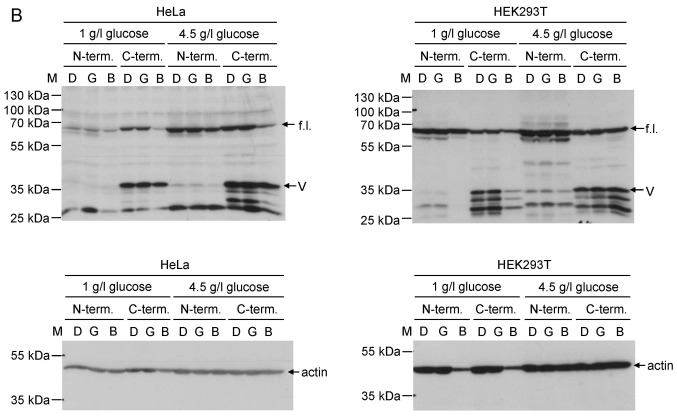
Effects of high glucose conditions on (pro)renin receptor [(P)RR] promoter activity and (P)RR isoform expresssion. (A) HeLa cells double-stably transfected with (P)RR promoter luciferase constructs were stimulated, after 24 h of starving, with prorenin for 24 h under different glucose concentrations (n=3 for each intervention). (B) HeLa and HEK293T wild-type cells, cultured under the indicated conditions, were transiently transfected with an expression vector encoding full-length RER C- or N-terminally fused with an EGFP-tag of 26 kDa (pEGFP-N1 or pEGFP-C3, respectively). (P)RR isoforms generated endogenously by post-translational processing were detected by Western blotting of total cellular proteins using an anti-GFP antibody. Anti-actin Western blotting served as loading control. M, molecular weight marker; D, 1% DMSO; G, 10 μM genistein; B, 0.1 μM bafilomycin; f.l., full-length (P)RR-GFP; V, V-ATPase domain of (P)RR fused to GFP.

Based on the pathophysiological role of the (P)RR in diabetic nephropathy, we examined its promoter regulation using different glucose conditions. High glucose increased basal (P)RR promoter activity ~3-fold (Fig. 3A). Strikingly, prorenin decreased (P)RR promoter activity only under high glucose conditions whereas an inverse response was observed in the cells cultured in a physiological glucose concentration (Fig. 3A).

Since the (P)RR is known to be expressed in different protein isoforms, we ascertained whether glucose and bafilomycin also affect these protein identities of the (P)RR. For this purpose, two expression vectors encoding full-length (P)RR N- or C-terminally fused to GFP, respectively, were transiently transfected into wild-type HeLa and wild-type HEK293T cells (Fig. 3B). Regarding specificity, the V-ATPase isoform can only be detected with the anti-GFP antibody after transfection of a C-terminally tagged (P)RR construct since the V-ATPase domain [~9 kDa (37)] within the full-length (P)RR [~38–39 kDa (23)] is located at the C-terminus. High glucose strongly increased full-length (P)RR in HeLa but not in HEK cells. Bafilomycin, in contrast to genistein, caused a relative shift from the full-length isoform to the V-ATPase isoform in HeLa cells due to a decreased abundance of the full-length form (Fig. 3B).

### Effects of glucose on prorenin glycosylation

**Figure 4:**
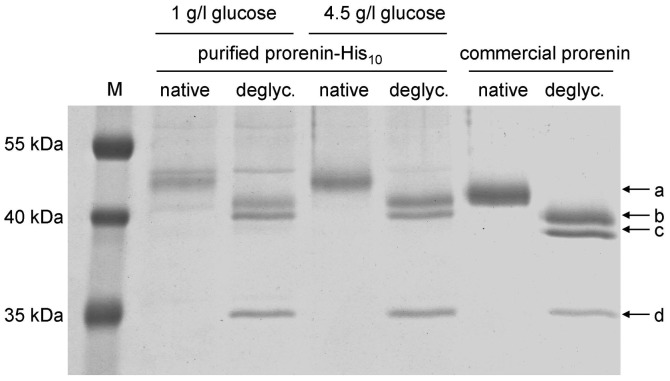
Effects of high glucose conditions on prorenin glycosylation. Prorenin purified from the supernatant of stably transfected Flp-In-293-HEK cells and a commercially obtained prorenin were deglycosylated (deglyc.) and resolved via sodium dodecyl sulfate-polyacrylamide gel electrophoresis (SDS-PAGE) followed by Coomassie staining. ‘Native’ indicates a mock incubation without glycosidase. M, molecular weight marker; a, completely glycosylated native or His-tagged prorenin; b, prorenin deglycosylated at one asparagine; c, prorenin deglycosylated at both asparagines; d, N-glycosidase F itself.

To ascertain whether high glucose directly affects the glycosylation of the ligand prorenin, we cultured HEK cells stably overexpressing prorenin in high or (relatively) low glucose medium (Fig. 4). The results revealed that HEK cells exhibit the ability to glycosylate prorenin. Furthermore, the two previously described N-glyosylation sites of prorenin (38) were confirmed as indicated by the double band in the presence of the glycosidase. Importantly, high glucose did not alter the glycosylation pattern of prorenin (Fig. 4).

### Effects of prorenin glycosylation on (P)RR promoter activity

**Figure 5:**
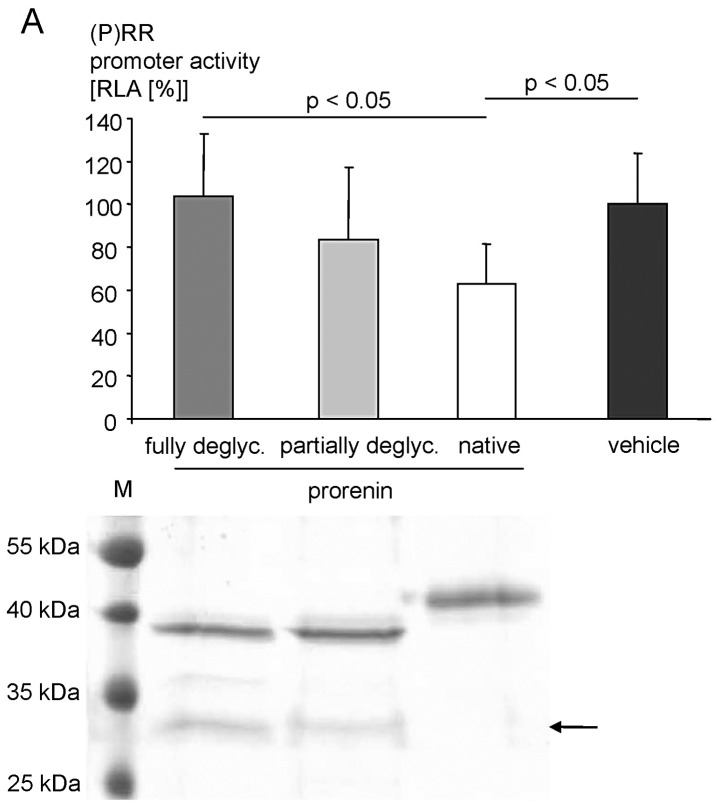

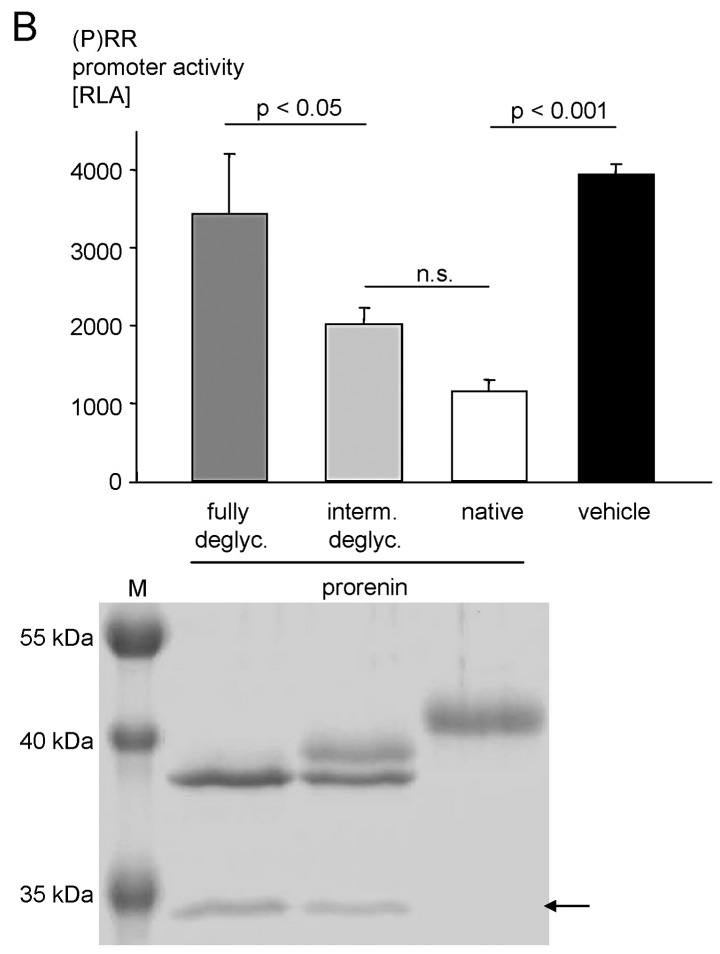
Deglycosylation of prorenin reduces its intrinsic activity. (A) Wild-type HeLa cells were transiently transfected with the (pro)renin receptor [(P)RR] promoter construct [−1; −1100]/pGL4.14 encoding firefly luciferase. Cotransfection of the phRL-null plasmid encoding humanised *Renilla* luciferase served for standardisation. Twenty-four hours after transfection, cells were starved for 24 h followed by stimulation with native or differently deglycosylated (deglyc.) prorenin (10 nM each; recombinant His_10_-tagged prorenin from Flp-In-HEK293 cells) for another 24 h. Relative luciferase activity (RLA) served as read-out. Data are based on three independent experiments (each n=3) and normalised to the vehicle group. (Lower panel) Coomassie-stained sodium dodecyl sulfate-polyacrylamide gel electrophoresis (SDS-PAGE) of native and differently deglycosylated prorenins used. The arrow indicates the N-glycosidase F itself. (B) Wild-type KELLY cells cultivated in 2 g/l glucose were transiently transfected with the (P)RR promoter construct [−1; −1100]/pGL4.14 encoding firefly luciferase (n=3). Cotransfection of the *Renilla*-encoding phRL-null plasmid served for standardisation. Twenty-four hours after transfection, cells were starved for 24 h followed by stimulation with native commercial (10 nM) or differently deglycosylated commercial prorenin (10 nM each) for another 24 h. n.s., non-significant. (Lower panel) Coomassie-stained SDS-PAGE of native and differently deglycosylated prorenins used. Intermediate (interm) deglyc.denotes the mixture of completely deglycosylated and partially deglycosylated prorenin molecules. The arrow indicates the N-glycosidase F itself.

We next aimed to ascertain whether deglycosylation impacts the ligand activity of prorenin in transiently transfected promoter assays with a sufficient transfection efficiency (as indicated by a firefly signal of the insertless control vector over 1,000 counts) and with a full deglycosylation of both asparagines (as confirmed by SDS-PAGE). As expected based on our previous results (23,24), native prorenin significantly repressed (P)RR promoter activity (Fig. 5A). Complete deglycosylation, as confirmed by SDS-PAGE, abolished this repressive effect (Fig. 5A).

Similar results were also obtained for neuronal KELLY cells. Native prorenin strongly repressed (P)RR promoter activity. Deglycosylation of both asparagines of prorenin abolished the ability to repress the (P)RR promoter, whereas a mixture of fully deglycosylated and partially deglycosylated (i.e., one of the two glycosylation sites is completely deglycosylated) prorenin exhibited intermediate repressive effects (Fig. 5B).

### Effects of glucose, genistein and bafilomycin on PLZF translocation

**Figure 6:**
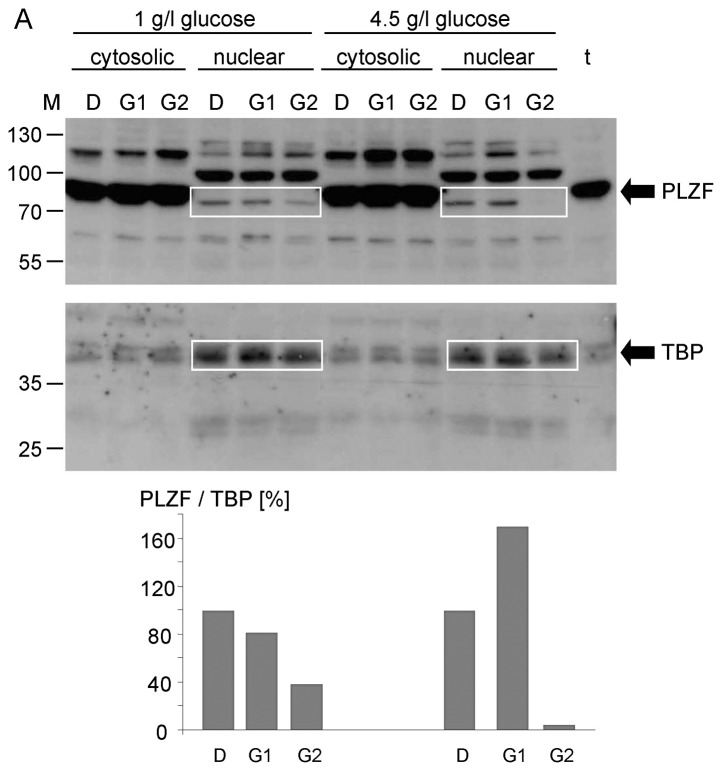

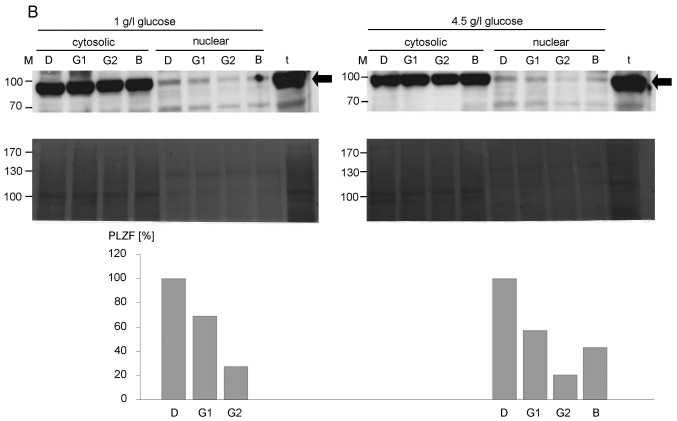
Genistein and bafilomycin affect nuclear promyelocytic leukemia zinc finger protein (PLZF) content. (A) HEK293T wild-type cells cultured under the indicated glucose concentrations were treated with 0.1% DMSO (D), 10 μM genistein (G1) or 100 μM genistein (G2) for 18 h before cell harvest for fractionated protein extraction. Total protein lysate (lane t) of HEK293T cells served as the positive control. Western blotting was performed using the anti-PLZF antibody and an anti-TBP antibody for standardisation. (Lower panel) Densitometric analysis of the nuclear protein fractions (as indicated by white rectangles in the western blot) is presented. According to the literature, the molecular weight of PLZF (arrow) is between 66 and 97 kDa (77). M, molecular weight marker in kDa. (B) Independent experiment as in (A) also including incubation with 1 μM bafilomycin (B) for 18 h before harvest. Coomassie staining (middle panel) served as the loading control.

Finally, we aimed to ascertain whether genistein and bafilomycin affect the nuclear translocation of PLZF at different glucose concentrations. Genistein (100 μM) (Fig. 6A) and bafilomycin (1 μM) (Fig. 6B) reduced the nuclear content of PLZF under physiological and high glucose conditions after 18 h in the HEK293T cells. This effect of genistein was not observed after 12 h in the HEK293T and KELLY cells (data not shown). Glucose concentration *per se* (i.e., under DMSO) appeared not to alter the PLZF content (Fig. 6).

### Cellular effects of the (P)RR sub-pathways

**Figure 7:**
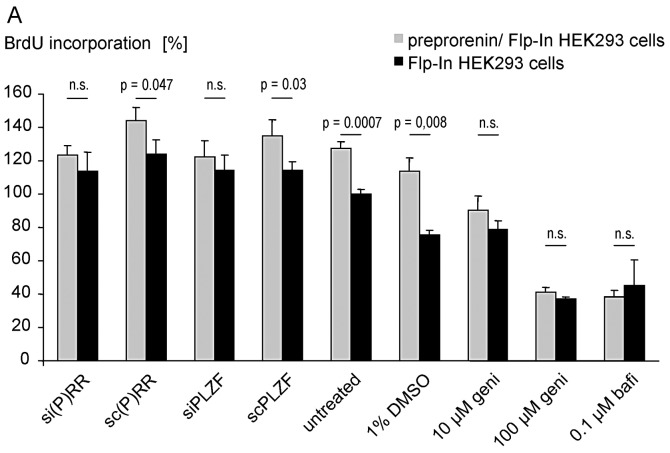

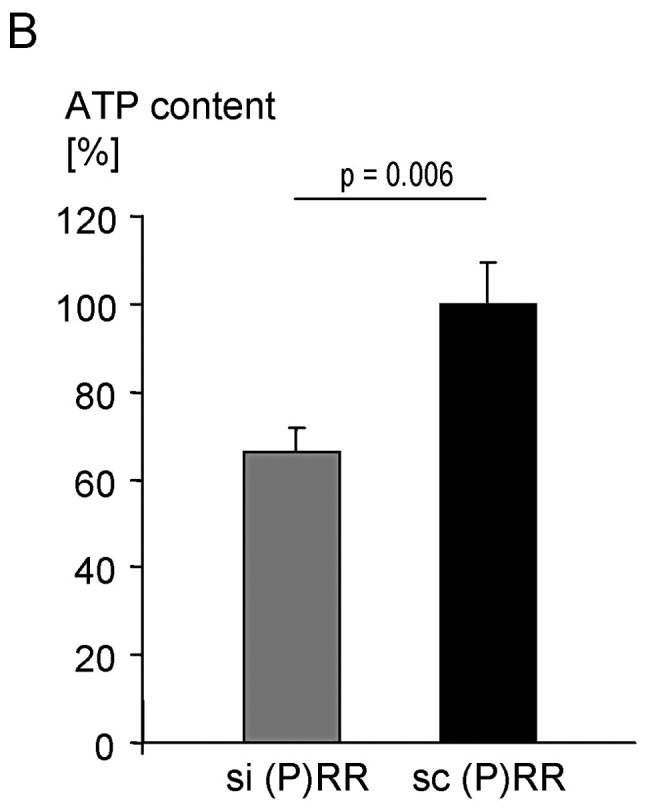

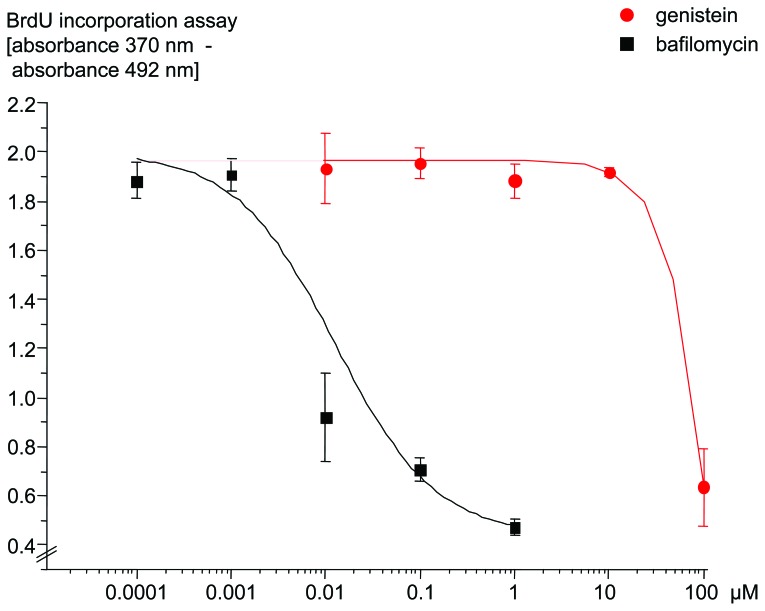

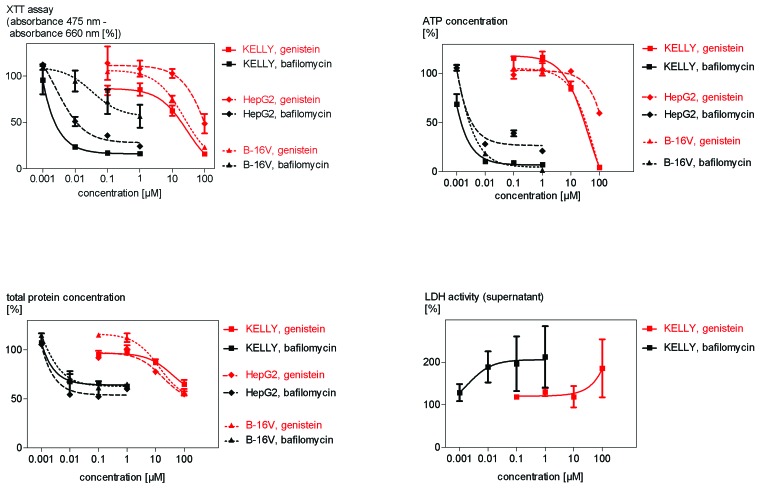
Cellular effects of the (pro)renin receptor [(P)RR] pathway. (A) The pro-proliferative effects of prorenin are mediated by (P)RR and promyelocytic leukemia zinc finger protein (PLZF). Flp-In HEK293 cells (cultured in 1 g/l glcuose) stably overexpressing prorenin or respective control cells were treated with siRNA against (P)RR [si(P)RR] or PLZF (siPLZF), respective scrambled control siRNAs (sc), genistein (geni) or bafilomycin A1 (bafi) for 48 h before harvest (n=3). Untreated (without transfection and without DMSO) Flp-In cells were set to 100%. n.s., non-significant. (B) Constitutive pro-proliferative effects of (P)RR in wild-type KELLY cells. Cell number was quantified in KELLY wild-type cells (2 g/l glucose) using an ATP-based assay (n=3) 48 h after transfection of siRNA against (P)RR [si(P)RR]. Scrambled siRNA [sc(P)RR] served as control. (C) Effects of genistein and bafilomycin on the proliferation in KELLY cells. KELLY wild-type cells (2 g/l glucose) were incubated with genistein or bafilomycin A1 for 48 h before harvest. The abscissa indicates the concentration. Proliferation was determined by BrdU incorporation assay (n=3). (D) Effects of genistein and bafilomycin on cell number in KELLY, HepG2 and B-16V cells and on the cytotoxic effect on KELLY cells. The indicated cell lines (2 g/l glucose) were incubated with genistein or bafilomycin A1 for 48 h. Mitochondrial dehydrogenase activity, an indicator of the number of metabolic active cell, was quantified by XTT assay in triplicates. Total cellular protein (n=3) and cellular ATP concentration (n=3) were measured using the Bradford assay and the Celltiter-Glo assay. Lactate dehydrogenase (LDH) activity in the supernatant was determined as a measure of cytotoxicity; 1% Triton X-100 served as a positive control (i.e., full kill) yielding an LDH activity of ~700% in KELLY cells. LDH data represent the mean of four independent experiments (each n=3). DMSO (1% final) was set to 100% in every experiment. Data values represented by the various symbols in all assays are the mean of triplicates ± standard deviation. Lines in all diagrams represent sigmoidal regression calculates as described in Materials and methods.

Regarding cellular effects of the (pro)renin-(P)RR-PLZF cascade, HEK cells stably overexpressing prorenin were analysed regarding proliferation using a BrdU assay (Fig. 7A). Compared to the non-overexpressing control cells, prorenin caused an increase in proliferation. This pro-proliferative effect was attenuated by siRNA against (P)RR, siRNA against PLZF and by pharmacological interventions using genistein or bafilomycin.

In addition, we examined the (pro)renin-independent cellular effects of the (P)RR. Repression of (P)RR expression by siRNA in wild-type neuronal cells in a system without (pro)renin supplementation significantly decreased cell number (Fig. 7B), whereas PLZF knockdown by siRNA had no effect (data not shown). Finally, the dose-dependent proliferative effects of genistein and bafilomycin were analysed in wild-type KELLY cells (Fig. 7C). Bafilomycin strongly decreased proliferation in contrast to genistein in a cellular system without incubation of recombinant or purified (pro)renin.

To analyse whether the altered proliferation is translated into different cell numbers, we performed XTT and ATP assay in addition to total protein measurements under the cell culture conditions as in Fig. 7B and C since these assays are indicative of total cell count (Fig. 7D). The shapes of the dose-response curves including the rightward shift between genistein and bafilomycin were observed in all of the assays and in the different cell types indicating that bafilomycin reduced the cell number through inhibition of proliferation. Furthermore, a minor contribution of direct cytotoxicity to the cell number-reducing effects of bafilomycin A1 was demonstrated using a LDH assay (Fig. 7D).

**Figure 8:**
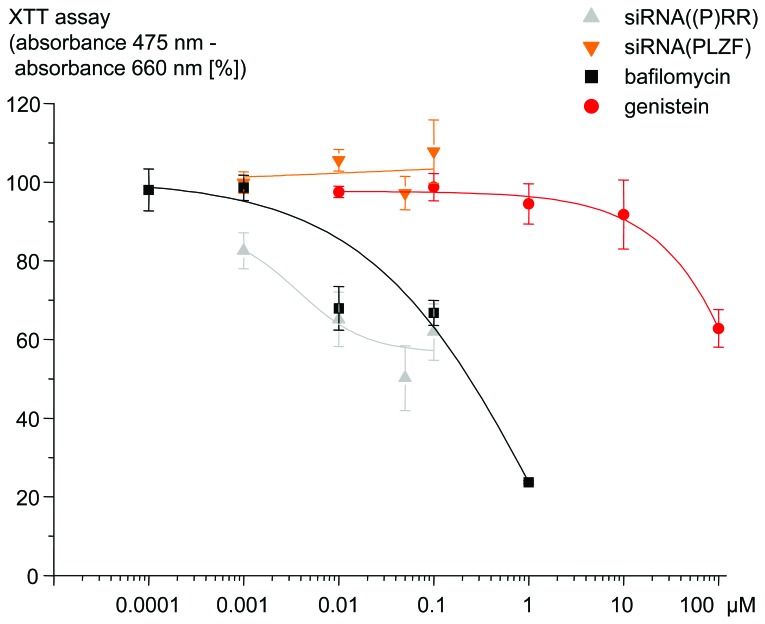
Correlation of small molecule and siRNA effects regarding cell number. HeLa cells double-stably transfected with the (pro)renin receptor [(P)RR] reporter constructs (4.5 g/l glucose) were incubated with different concentrations of genistein and bafilomycin for 48 h. In addition, siRNA against (P)RR or promyelocytic leukemia zinc finger protein (PLZF) was applied once for 48 h at the indicated concentrations: 1, 10, 50 and 100 nM, respectively. The number of viable cells were determined by XTT assay. The data for the small molecule and siRNA interventions were standardised to DMSO or scrambled siRNA, respectively, i.e., a value of 100 on the ordinate indicates an effect size that equals 1% DMSO or scrambled siRNA. All measurements are triplicates, and the error bars denote standard deviation.

Finally, we examined whether small molecule interventions using genistein or bafilomycin and gene silencing approaches using siRNA exhibit similar effects. Affecting the (P)RR by either bafilomycin or siRNA, in the absence of (pro)renin, both strongly reduced cell number in a concentration-response relationship (Fig. 8). In contrast, affecting PLZF by either exposure to genistein or siRNA, in the absence of prorenin, did not alter the cell number with the exception of 100 μM genistein (Fig. 8).

**Figure 9:**
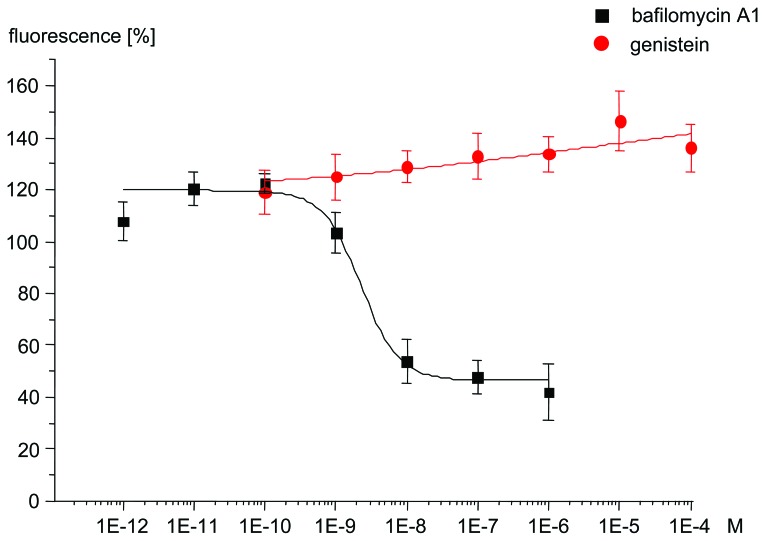
Differential effects of bafilomycin and genistein on lysosomal/peroxisomal pH. HeLa wild-type cells (4.5 g/l glucose) were incubated with the indicated concentrations of bafilomycin A1 or genistein for 2 h before Lysotracker fluorescence emission was measured (n=6/concentration of each compound). Indicated concentrations are defined as follows: 1E-12 M =10^−12^ M and so forth.

Concerning the intracellular phenotypic impacts of genistein and bafilomycin, we determined intravesicular pH regulation using Lysotracker fluorescence dye in HeLa cells. To exclude effects on cell number, incubation of these substances was restricted to 2 h. Fig. 9 indicates that bafilomycin increased lysosomal/peroxisomal pH with an EC_50_ of ~2 nM whereas genistein had no effect.

## Discussion

There are three major findings of this study regarding the (P)RR signal transduction cascade. First, ligand glycosylation is a crucial determinant of intrinsic activity. Second, glucose concentration affects (P)RR signalling at different levels. Third, the (P)RR exhibits constitutive, PLZF-independet pro-proliferative effects.

In the present study, we demonstrated that the steady-state glucose concentration does not affect the glycosylation pattern of prorenin (Fig. 4), in contrast to HbA1c glycation (i.e., non-enzymatic glycosylation) by glucose (39). The glycosylation pattern itself is crucial for the ligand activity of prorenin since deglycosylation abolishes the effect of prorenin on the (P)RR signal transduction (Fig. 5). Furthermore, a mixture of fully deglycosylated and partially deglycosylated (i.e., one of the two glycosylation sites was completely deglycosylated) prorenin is sufficient to mediate half-maximal intrinsic activity (Fig. 5B). To the best of our knowledge, this is the first report demontrating that the intrinsic activity of prorenin depends on its glycosylation.

In contrast to the glucose-independent ligand glycosylation, high glucose conditions increased the basal (i.e., without prorenin stimulation) (P)RR activity as measured by promoter assay, and reversed the ability of prorenin regarding (P)RR activation (Fig. 3A). This increased basal (P)RR receptor activity under high glucose was likely caused by an increased receptor expression based on the observation that glucose induced (P)RR at the protein level in HeLa cells (Fig. 3B). The increased full-length (P)RR protein expression appeared not to alter the (P)RR isoform ratio in HeLa cells (Fig. 3B).

The strong upregulation of full-length (P)RR protein expression in HeLa cells cultivated in high glucose conditions observed in this study is consistent with previous studies which demonstrated an upregulation of (P)RR mRNA and protein in hearts (21) as well as kidneys (22) of diabetic rats, of (P)RR protein in kidneys of patients with diabetic nephropathy (40), and of (P)RR mRNA and protein in rat mesangial cells by glucose (41). Mechanistically, NF-κB, AP-1 and Sp1/Sp3 appear to be involved in the (P)RR promoter regulation by high glucose (42). The authors showed that an exchange of c-jun, c-fos, NF-κB p65 and NF-κB p50 on *cis*-elements of the (P)RR promoter mediated the glucose responsiveness of this gene, indicating a complex promoter regulation in which more than one transcription factor was involved. Furthermore, dissocation of V-ATPase subunits was found to be promoted by low glucose levels (43) which in turn may affect the regulation of the (P)RR gene, since it encodes an essential accessory protein of V-ATPases (44).

Focusing on the regulatory levels, bafilomycin and genistein increased (P)RR promoter activity and (P)RR mRNA (Fig. 2A–D). Bafilomycin but not genistein decreased exogenous (i.e., under control of a CMV promoter) full-length (P)RR protein in HeLa cells (Fig. 3B). Concerning endogenous (P)RR, soluble (P)RR was strongly increased by bafilomycin in the total lysates and in the supernatant (Fig. 2E and F). This clearly increased s(P)RR expression may be reflected by an increased translation of full-length (P)RR followed by direct processing into the soluble isoform and a rapid degradation of the V-ATPase-associated isoform. This would also explain why the full-length (P)RR protein expression was almost unaltered by bafilomycin (Fig. 2E) and is consistent with a recent conclusion by Fukushima *et al,* that the elevation of plasma s(P)RR level may indicate an upregulation of the full-length form (45).

Here, we were able to confirm the previous data of our group (23,24) that revealed that prorenin mediates pro-proliferative effects via the (P)RR-PLZF axis (Fig. 7A) which is also consistent with a microarray analysis linking (pro)renin stimulation with a gene signature associated with cardiac hypertrophy (20). Furthermore, research indicates that (pro)renin increases DNA synthesis (46) and proliferation (47) of vascular smooth muscle cells, and that prorenin can increase protein and DNA synthesis in cardiomyocytes underlying myocyte hypertrophy and proliferation (48). In addition, it was found that prorenin increased the proliferation of endothelial cells and that melanoma xenografts stably transfected with prorenin had an increased tumour growth *in vivo* compared to mock controls likely involving the (P)RR since an angiotensin AT1 receptor blocker was ineffective *in vitro* (49).

**Figure 10:**
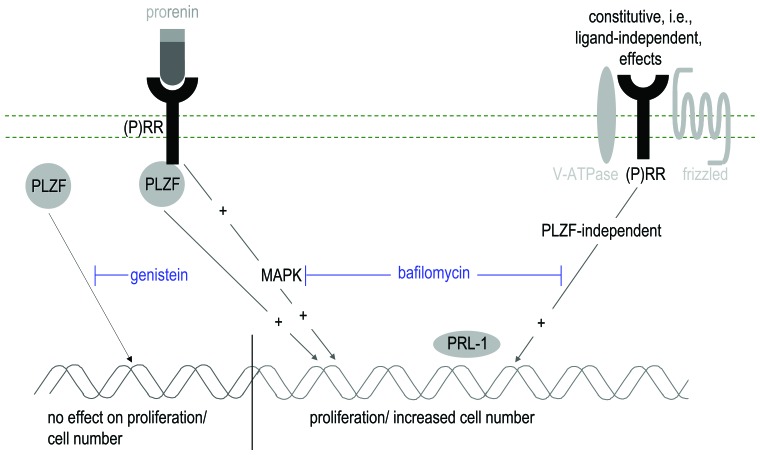
The prorenin receptor [(P)RR] mediates constitutive promyelocytic leukemia zinc finger protein (PLZF)-independent as well as prorenin-mediated PLZF-dependent pro-proliferative effects. The pro-proliferative effect of the prorenin-(P)RR-PLZF axis was previously demonstrated by our group (24) whereas the constitutive functions of the (P)RR were analysed in the present study. MAPKs (78), the Wnt pathway (79) and V-ATPases (80) are known to mediate proliferation in general and likely contribute to pro-survival/pro-proliferative effects of the (P)RR (21,51,81,82). In addition, the phosphatase PRL-1 may be involved in the regulation of proliferation downstream of the (P)RR (1). The inhibitory effects of bafilomycin on the prorenin-induced activation of MAPKs have been shown by Advani *et al* (29). Genistein inhibits basal (data obtained in this study) and angiotensin II-induced (33) nuclear translocation of PLZF.

Our data obtained in neuronal, melanoma, hepatoma and epithelial cells in the absence of stimulation with the ligand (pro)renin indicate that the (P)RR exerts additional constitutive, cell type-independent pro-proliferative/pro-survival effects (Fig. 7C and D). These effects are independent of PLZF as indicated by our experiments using siRNA against PLZF or genistein. This is in contrast to the essential role of PLZF in (pro)renin-induced pro-proliferation as discussed above (Fig. 10). This constitutive function of the (P)RR is consistent with the phenotype of the cardiomyocyte-specific (P)RR knockout which is characterised by cardiac cell death (50) and also with the phenotype of the podocyte-specific (P)RR knockout which is characterised by non-apoptotic podocyte cell death (51) since both phenotypes can be explained by a V-ATPase dysfunction. Moreover, repression of (P)RR expression by siRNA reduced the viability of cultured cardiomyoblasts in an experimental setting without (pro)renin incubation (21). The role of the constitutive (P)RR function in cellular survival is also supported by the central nervous system necrosis observed in zebrafish with (P)RR gene mutagenesis (52) but also in zebrafish with genetic alterations in different V-ATPase subunits (30). Furthermore, the ligand renin is only expressed in mammalian and nonmammalian vertebrates (53) but not in invertebrates such as *C. elegans*, which is not viable when (P)RR is lacking (27). The importance of the constitutive activity is further supported by the fact that at least plasma (pro)renin levels and, therefore, even more (pro)renin concentrations in cell culture medium with a serum content of 10%, are too low to be of biological relevance with respect to (P)RR activation (54).

The extent to which (pro)renin-independent effects are mediated by the Wnt pathway, considering the essential role of the (P)RR in its signalling (26), and/or by the V-ATPase isoform remains to be elucidated. Consistent with a role of Wnt in the basal (i.e., ligand-independent) effects of the (P)RR observed here, it was shown that (P)RR function within the Wnt pathway is renin-independent (26).

The overall (i.e., ligand-dependent and/or ligand-independent) role of the (P)RR in cellular survival is also illustrated by the observation that embryonic stem (ES) cells deficient in the (P)RR gene are incompatible with the development of chimeric mice when injected into blastocysts (51,55) and by the likely involvement of the (P)RR in the growth of glioma cells (56).

The present study is the first to simultaneously address the effects of the small molecules genistein and baflomycin. Genistein is a phytoestrogen known to interact with estrogen receptors α (ERα) and ERβ leading to activation of ER responsive genes (57,58). Furthermore, genistein can inhibit tyrosine kinases (59) and aspects of Wnt signalling (60) in addition to its effects on histone modifications and DNA methylation (i.e., epigenetic modulations) (61) as well as on NF-κB (62) and Smad (63) signal transductions. In the context of our data, it is important to note that genistein inhibits the nuclear translocation of PLZF (33). We also observed that genistein inhibited nuclear translocation of PLZF in HEK293T cells indicating the contribution of this genistein-mediated mechanism in our experiments. Since HeLa-S3 cells do not express ERα and ERβ receptors (data not shown) the effects of genistein on this cell type are non-ER-mediated. Similar to our data that genistein does not significantly inhibit cell growth, the MTT assay-determined IC_50_ value of genistein in a panel of cancer cell lines was in the two-digit micromolar range (64). Consistently, a recent clinical phase II trial indicated that genistein did not increase the survival of pancreatic cancer patients (65).

Bafilomycin A1 is a specific V-ATPase inhibitor which can inhibit the ligand- (i.e., prorenin- and renin-) dependent (29) and Wnt-associated (26) signal transduction of the (P)RR.

In accordance with our data, bafilomycin A1 was found to decrease the growth of different tumour cell lines *in vitro*, with an IC_50_ of 5 nM regarding cellular viability of pancreatic cancer cells (68), as well as xenograft growth *in vivo* (66–68). Nevertheless, its toxicity excludes its use in clinical trials (69,70).

Concerning cardiovascular indications, it was recently demonstrated that genistein reduced proteinuria, albuminuria and glomerular deposits in streptozotocin-induced diabetic mice (71), similar effects as observed with the anti-prorenin decoy peptides discussed above (3). In addition to these beneficial effects, genistein was found to protect pancreatic β cells from high glucose-induced apoptosis (72). In contrast, bafilomycin A1 reduced pancreatic islet size and impaired glucose tolerance in animal models (73,74) hypothetically linking this bacterial toxin to the development of type I diabetes (75).

Based on our data, we conclude that genistein is a small molecular mimetic of siRNA against PLZF, whereas bafilomycin exerted similar effects as siRNA against (P)RR for the following reasons. First, genistein, bafilomycin as well as siRNAs against PLZF or (P)RR all increased (P)RR promoter activity (Figs. 1 and 2B). Secondly, neither PLZF silencing by siRNA nor genistein exerted significant effects on cell number (Fig. 8). In all our cellular phenotypic assays regarding total protein concentration, cell number and proliferation, genistein had no effects except at the highest (100 μM) concentration used (Fig. 7C and D). Since this concentration is far beyond the EC_50_ (Fig. 2B), this indicates putative unspecific effects at 100 μM, which is consistent with the threshold of 5 μM genistein regarding a non-physiological *in vitro* concentration (76). In contrast, both siRNA against (P)RR and also bafilomycin similarly reduced cell number (Fig. 8). Third, other groups have shown that wild-type podocytes treated with bafilomycin A1 are characterised by similar morphologic and pH changes compared to podocytes with (P)RR deletion (51).

We further conclude that (P)RR and PLZF functions are not identical, despite a similar impact on promoter feedback (Fig. 1) and a similar effect on prorenin-induced proliferation (24), for the following reasons. First, as discussed above, (P)RR affected cell number/proliferation in contrast to PLZF. Second, genistein and bafilomycin had distinct effects on (P)RR isoform expression (Fig. 3B). Third, bafilomycin, as expected, increased lysosomal/peroxisomal pH as indicated by a reduction in Lysotracker fluorescence emission in contrast to genistein (Fig. 9).

To conclude, our data indicate that the (P)RR does not only exert angiotensin II-independent (2, 23) but also (pro)renin-independent (i.e., constitutive) effects. By employing dose-response analyses and various cellular assays, the present study is the first detailed description of the constitutive, pro-proliferative/pro-survial actions of this receptor. Furthermore, the novel finding that glycosylation of prorenin is crucial regarding its ability to initiate a signal transduction at the (P)RR was demonstrated.
